# Supplementary material for: The influence of demographic and socio-economic factors on non-vaccination, under-vaccination and missed opportunities for vaccination amongst children 0–23 months in Kenya for the period 2003–2014
Source: PLOS Glob Public Health. 2024 May 30;4(5):e0003048. doi: 10.1371/journal.pgph.0003048 (PMC11139289; doi:10.1371/journal.pgph.0003048)
Supplement: Attachment — Submitted filename: PGPH-D-24-00200_R1_Lazare_Review.pdf [file pgph.0003048.s004.pdf]

Peer Review manuscript, by Lazare M'BOUNGOU.

1. Manuscript number: PGPH-D-24-00200R1

2. <u>Article title reviewed</u>: *The influence of demographic and socio-economic factors on non-vaccination, under vaccination and Missed Opportunities for Vaccination amongst children 0-23 months in Kenya for the period 2003-2014.*

3. <u>Outputs of the review</u>:

Very interesting research topic: classic but complex due to the number of outcome variables (non-vaccination, under-vaccination and missed opportunities vaccination) and the number of survey repetitions (2003, 2008/09 and 2014).
The study has been conducted rigorously and the parallelism of forms has been respected between the periods.
The authors have methodically integrated the observations of my peer reviewers, which significantly improved the quality of the first manuscript. However, the current version of the manuscript would gain more if the authors could consider the additional comments below:

Methods section (2/2):

1. We suggest the authors to explain how the Littoral province was chosen as the reference modality for the Province variable (It's not necessary to explain the choice of reference modality for each of the other variables).

2. The study area needs to be completed with more details. Indeed, the manuscript specifies: "Study area This study covered the entire 47 counties in Kenya. The KDHS surveys were national level surveys, which in 2003 and 2008/09 were conducted across all the former eight Provinces of Kenya and in 2014, was conducted in all the 47 counties in line with the new administrative units". This means changes in the administrative division between 2009 and 2014. If so, the authors are invited to explain or specify that the changes in the administrative division have not affected the provinces (in number and name) between 2003 and 2014.

Results section (2/2):

1. We recommend that the authors present the socio-demographic characteristics of the study participants in the form of tables and/or graphs (numbers, frequencies, means, standard deviation, median, etc. depending on the characteristic and/or the outcome variable).

2. We know that for a given variable, OR (reference modality) = 1. I suggest that the authors replace REF by 1 in each table (Tables 1, 2 and 3).

Another comment (1/1):

1. I didn't see any appendices in the current version of the manuscript. If you have not already done so, it would be advisable to include the following: the bivariable logistic regression and the multivariable logistic regression model initial.
