# Supplementary material for: The influence of demographic and socio-economic factors on non-vaccination, under-vaccination and missed opportunities for vaccination amongst children 0–23 months in Kenya for the period 2003–2014
Source: PLOS Glob Public Health. 2024 May 30;4(5):e0003048. doi: 10.1371/journal.pgph.0003048 (PMC11139289; doi:10.1371/journal.pgph.0003048)
Supplement: Attachment — Submitted filename: Response to Reviewers.docx [file pgph.0003048.s002.docx]

March 05, 2024

Dr Collins Otieno Asweto, PhD,

Academic Editor,

PLOS Global Public Health

**Response to Reviewers - PGPH-D-24-00200**

**The influence of demographic and socio-economic factors on non-vaccination, under-vaccination and Missed Opportunities for Vaccination amongst children 0-23 months in Kenya for the period 2003-2014.**

We extend our sincere appreciation for your meticulous review of our manuscript and the invaluable insights you and the reviewer have provided. We have carefully considered both your comments and recommendations, and we respectfully offer our responses as in the accompanying responses to reviewer’s document.

In conjunction with the document addressing each raised point from both your esteemed self and the esteemed reviewer(s), we have attached a marked-up version of our manuscript, highlighting the revisions made, alongside an unmarked version of the revised manuscript for your perusal.

**Reviewer #1**: I found the manuscript to be well-written, comprehensive, and methodologically sound. The authors have conducted a thorough investigation into demographic and socio-economic determinants of immunization coverage gaps in Kenya. The research question is clearly articulated and addresses an important issue in public health. The methodology is rigorous and appropriate for the research objectives, and the results are well-presented and supported by the data analysis. Also, the discussion section provides insightful interpretations of the findings and contextualizes them within the existing literature. However, there is a need for citation at the end of line 416: “The preference for home deliveries might be associated with lower health literacy or socio-economic challenges among mothers, impacting their awareness and ability to prioritize timely immunizations” because evidence exists. Overall, the manuscript adheres to the journal's guidelines and standards for publication.

**Response: We appreciate your positive review of our work and have made the appropriate citation as guided on the relevant section of the manuscript. We have cited 3 relevant papers numbered 38-40.**

**Reviewer #2**: This is a promising article in an important topic with potential for application to public health policies in Kenya. However, in its current format, the manuscript does not meet the criteria for a scientific publication. The inexperience of the researchers is apparent in several aspects, from the design of the study to the quality of the manuscript. This article would benefit from a major review by a experienced epidemiologist; commenting in all the areas where the manuscript benefits from improvement is beyond the scope of this review. Some of the fundamental flaws, which are not addressed, are obviously apparent. For example, why did the authors not combine the three waves of DHS, which is the standard and preferable approach when analysis DHS data? Combining the three waves of DHS would increased the sample size, therefore increasing power to detect smaller effects, and the inclusion of an indicator variable for the wave would indicate temporal trends in the outcome, from one wave of DHS to the other. The "methods" section is superficial and does not describe, nor address, fundamental questions related to a epidemiological study such as this one. The absence of relevant references is present throughout the manuscript. Overall, this manuscript would benefit from the collaboration with an experienced epidemiologist who could point out the extensive opportunities for improvement.

**Response**: We appreciate your thoughtful evaluation of our manuscript and your recognition of its potential significance in the field of public health in Kenya. We understand your concerns regarding the current format of the manuscript and take your feedback seriously.

We would like to address some of the points you raised. Firstly, we acknowledge your suggestion regarding the combination of the three waves of DHS data. This is exactly what we did. Our study was specifically designed to compare differences between the three survey points, which we believe is valuable in understanding temporal trends.

Regarding the depth of our methods section, we respectfully disagree with your assessment. We believe our methods are robust and adequately describe the procedures employed in our study.

Regarding the issue of references, we have carefully selected relevant citations to support our claims and have incorporated additional references in response to an initial feedback by reviewer 1. We are confident that the current manuscript provides a comprehensive review of the existing literature relevant to our study.

We understand your recommendation for collaboration with an experienced epidemiologist, and while we appreciate the value of such collaboration, we would like to highlight that our research team comprises seasoned epidemiologists with extensive experience in the field. We are confident in the quality of our work and believe that we have produced a manuscript worthy of publication in PLOS Public Health Journal.

Once again, we thank you for your time and constructive criticism.

**Reviewer #3**: This was a quantitative study that analyzed secondary data from longitudinal repeated annual and national cross-sectional surveys obtained from the Kenya demographic and health surveys conducted in 2003-2014. These data were analyzed to determine Non-vaccination, under-vaccination and missed opportunities for vaccination which had posed significant challenges to the benefits of vaccination in children 0-23months in Kenya.

The authors have presented an insightful study from a massive research with results, statistics and analysis performed to a high standard with sufficient details presented. The conclusions drawn were supported by the data presented in the research work. I commend the authors for a diligent and painstaking research work presented here. It captures the essence of a good quantitative study.

**Response: We similarly appreciate your positive review of our work and are confident it will add to the body of knowledge in the immunization space.**

**Reviewer #4**: The immunization rates in Kenya throughout the time period under study were expertly highlighted by the writers. However, I have made the following observations that need correction:

1. There were numerous typographical errors that need attention. See, for instance, pages 222, 280, 365, 405, etc. In several lines, there were also missing punctuation marks. A thorough editing will address this oversight.

**Response: We appreciate your keenness in picking these typographical errors and as a result, have endeavored to thoroughly review and correct all typographical errors in our paper. The errors noted here have also been corrected.**

2. The writers used only one citation when they wrote, "...other studies cite that..." The wording should indicate if they are relying just on one citation to support their claim.

**Response: We have made edits to all relevant areas to indicate the appropriate evidence supporting our claims. These include in lines 280, 283, 284, 313, 338, 346, 347, 350, 355, 374 and 375.**

3. The language syntax used in the results presentation should be adjusted to accurately represent the message that the authors intended to convey. Example, lines 187–193 may cause confusion for the reader on the relationship between the mother's primary school education and non-vaccination.

**Response: We have edited the statements on lines 187-193 to reflect a likelihood of non-vaccination in the various categories of mothers’ education and Province.**

We appreciate your consideration of our manuscript for publication in PLOS Global Public Health and look forward to the opportunity to contribute to the dissemination of knowledge in the field. We are eagerly anticipating your endorsement of our manuscript for publication. Your guidance and insights have been invaluable in enhancing the quality and rigor of our work.

Best regards,

Christopher Ochieng’ Odero,

P. O. Box 51048 – 00200,

Nairobi.

E: [christodero@gmail.com](mailto:christodero@gmail.com)
