# Supplementary material for: The influence of demographic and socio-economic factors on non-vaccination, under-vaccination and missed opportunities for vaccination amongst children 0–23 months in Kenya for the period 2003–2014
Source: PLOS Glob Public Health. 2024 May 30;4(5):e0003048. doi: 10.1371/journal.pgph.0003048 (PMC11139289; doi:10.1371/journal.pgph.0003048)
Supplement: Attachment — Submitted filename: review -sarashwati Giri.docx [file pgph.0003048.s003.docx]

**Materials and methodology**

**Strength:**

**- The sample size calculation is comprehensive, considering various factors like design effect and response rates, ensuring robust estimation.**

**Recommendations:**

**- Address potential biases and limitations in the methodology to strengthen validity.**

**- Provide contextual interpretation of results and ensure consistency in reporting across different years for better comparison.**

**Results:**

**- Tables are well-arranged and interpreted, demonstrating meticulous attention to detail and enhancing the study's credibility.**

***Discussion:***

**- Clear, accurate, and insightful discussion on factors influencing childhood vaccination in Kenya.**
