# Supplementary material for: The influence of demographic and socio-economic factors on non-vaccination, under-vaccination and missed opportunities for vaccination amongst children 0–23 months in Kenya for the period 2003–2014
Source: PLOS Glob Public Health. 2024 May 30;4(5):e0003048. doi: 10.1371/journal.pgph.0003048 (PMC11139289; doi:10.1371/journal.pgph.0003048)
Supplement: Attachment — Submitted filename: Response to reviewers.docx [file pgph.0003048.s005.docx]

April 10, 2024

Dr Collins Otieno Asweto, PhD,

Academic Editor,

PLOS Global Public Health

**Response to Reviewers - PGPH-D-24-00200R1**

**The influence of demographic and socio-economic factors on non-vaccination, under-vaccination and Missed Opportunities for Vaccination amongst children 0-23 months in Kenya for the period 2003-2014.**

We extend our sincere appreciation to you and the reviewers for the meticulous review of our manuscript and the invaluable insights provided. We have carefully considered both your comments and recommendations, and we respectfully offer our responses as in the accompanying responses to reviewer’s document.

In conjunction with the document addressing each raised point from both your esteemed self and the esteemed reviewers, we have attached a marked-up version of our manuscript, highlighting the revisions made, alongside an unmarked version of the revised manuscript for your perusal.

**Reviewer 5:**

**Materials and methodology**

**Comment: Strength:**

- **The sample size calculation is comprehensive, considering various factors like design effect and response rates, ensuring robust estimation.**

**Response**: We are pleased that you found our sample size calculation comprehensive, taking into account various factors such as design effect and response rates to ensure robust estimation.

**Comment: Recommendations:**

- **Address potential biases and limitations in the methodology to strengthen validity.**

**Response**: We appreciate the reviewer's thorough examination of our study methodology and their request to address potential biases and limitations. We recognize the importance of ensuring the validity of our findings and have taken into account several inherent limitations in our study design and data sources.

As previously noted in our manuscript, relying on existing data from the Kenya Demographic and Health Surveys (KDHS) conducted between 2003-2014 inherently limits the inclusion of detailed variables and may introduce errors from the original data collection process. Additionally, the binary categorization of MOV status, determined by an algorithm, may oversimplify the complex nature of MOV patterns. Furthermore, the cross-sectional design of our study prevents the establishment of causal relationships between demographic and socio-economic factors and vaccination outcomes.

We also acknowledge potential biases, such as social desirability in self-reported data, and unobserved social and cultural factors, which may impact the reliability of our results. Changes in healthcare policies and contextual factors over time, along with missing data and ethical considerations, further contribute to the limitations of our study.

While we have endeavored to mitigate these limitations through rigorous analytical methods and transparent reporting, we recognize that they remain inherent to our study design. We have revised the language in our manuscript to emphasize the importance of interpreting our findings within the context of these constraints and to suggest avenues for future research or enhancements in data collection methods that may address these limitations. See page 33 lines 473 to 478.

**Comment: Provide contextual interpretation of results and ensure consistency in reporting across different years for better comparison.**

**Response**: We have taken your advice to heart, and whilst we believe our results have been placed in appropriate context, we have once again reviewed the interpretation of our results. We believe that these results offer an explanation and emphasizes the nuances of childhood vaccination in the Kenyan context across the different years.

**Comment: Results:**

- **Tables are well-arranged and interpreted, demonstrating meticulous attention to detail and enhancing the study's credibility.**

**Response**: We are also happy that you found our tables well-arranged and interpreted, reflecting our meticulous attention to detail. Ensuring the clarity and accuracy of our results is paramount to us, and we appreciate your acknowledgment of our efforts in this regard.

**Comment: Discussion:**

- **Clear, accurate, and insightful discussion on factors influencing childhood vaccination in Kenya.**

**Response**: Your feedback on the discussion section is encouraging, and we are pleased that you found our analysis clear, accurate, and insightful.

**Reviewer #6:**

**Comment: Introduction: It would be good to add similar studies conducted within the region (Talk about non-vaccination, under vaccination and Missed Opportunities for Vaccination in East African countries, if there are any.**

**Response**: We have considered this recommendation and included some regional level studies on this topic, identifying the gaps they have and the reason why our study is relevant. Please refer to page 4, lines 86 to 90.

**Comment: Add the reference for this paragraph>>>>In Kenya, the National Vaccines and Immunization Program (NVIP) recommends that a child receives Bacillus Calmette–Guérin (BCG) and oral polio vaccine (OPV) at birth; pentavalent vaccine, OPV, rotavirus vaccine and pneumococcal conjugate vaccine (PCV) at 6, 10 and 14 weeks, vitamin A at 6 months and measles vaccine at 9 months. Routine vaccines are delivered for free at public health facilities, with outreach services conducted in hard-to-reach communities. Through routine vaccination the government aims to achieve equitable access to health services, especially for children.**

**Response**: In order to provide more clarity and understanding, we have added appropriate references in the suggested paragraph. We have cited the Kenya Ministry of Health National Immunization Policy Guidelines. Please refer to page 3, lines 61 & 64.

**Comment: Methods: Why were all the variables from the bivariate logistic regression included in the multivariate logistic regression? As it is better to include only those that are statistically significant, it would be good if you could provide/ add a clear explanation of why you have chosen to include all the variables.**

**Response**: We have considered your suggestion and provide more supportive information.

For all comparisons, results with p-values <0.2 were included in a final multivariable logistic regression. This allowed for a more comprehensive exploration of potential factors influencing the outcome variables, besides those with statistically significant p-values (≤0.05), ensuring that all relevant variables are considered while still maintaining the rigor of statistical significance in the final model. We have included this statement on page 8, lines 176 to 182.

**Comment: Results: In Table 3, the variable Protestant/other Christian has been made significant for the 2003 data while the CI crosses 1: 0.74 (0.41-1.33). Is it a mistake or there is a reason behind?**

**Response**: Thank you for picking this up. It was erroneously marked as significant. We have made the necessary correction on the table. Since this was a COR, we did not provide an explanation on the text as we did with the corresponding AOR.

**Reviewer #7:**

**Comment: Very interesting research topic: classic but complex due to the number of outcome variables (non-vaccination, under-vaccination and missed opportunities vaccination) and the number of survey repetitions (2003, 2008/09 and 2014).
The study has been conducted rigorously and the parallelism of forms has been respected between the periods.**

**Response**: We are pleased that you found our research topic interesting, despite its complexity. Your acknowledgment of the rigor with which the study was conducted and the maintenance of parallelism across survey periods is greatly appreciated. We recognize the challenges inherent in examining multiple outcome variables—non-vaccination, under-vaccination, and missed opportunities vaccination—across different survey years (2003, 2008/09, and 2014). Our aim was to provide a comprehensive analysis of these variables to gain a nuanced understanding of childhood vaccination patterns over time in our study population. We are pleased that you found our methodology rigorous and that we maintained consistency in the survey instruments across the various periods. Ensuring parallelism in data collection methods is crucial for enabling meaningful comparisons across time points, and we are glad that our efforts in this regard were noted.

**Comment: The authors have methodically integrated the observations of my peer reviewers, which significantly improved the quality of the first manuscript. However, the current version of the manuscript would gain more if the authors could consider the additional comments below:**

**Methods section (2/2):**

**We suggest the authors to explain how the Littoral province was chosen as the reference modality for the Province variable (It’s not necessary to explain the choice of reference modality for each of the other variables).**

**Response**: Even though the reference variable for Coastal Province was randomly selected, there are key unique factors to note that also support its selection as a reference variable. The inhabitant population has maintained traditional cultural beliefs and practices that may impact on their access to health care services in health facilities. These include non-exposure of newborns before they reach 40 days’ post-partum. In addition, they are predominantly of the Muslim religion. Further, girls are married off at an earlier age, tend not to have completed formal education and with no formal occupation.

**Comment: The study area needs to be completed with more details. Indeed, the manuscript specifies: “Study area This study covered the entire 47 counties in Kenya. The KDHS surveys were national level surveys, which in 2003 and 2008/09 were conducted across all the former eight Provinces of Kenya and in 2014, was conducted in all the 47 counties in line with the new administrative units”. This means changes in the administrative division between 2009 and 2014. If so, the authors are invited to explain or specify that the changes in the administrative division have not affected the provinces (in number and name) between 2003 and 2014.**

**Response**: We have provided more details on the study area section of the manuscript to clarify this point. Please refer to page 5, lines 108 to 113 for this explanations. The changes in the administrative units (Counties) were implemented to enhance administration and service delivery, including for health among others. The administrative units (Counties) were curved out of the former 8 provinces in Kenya. In our analysis, we have grouped the Counties as per the 8 former provinces to ensure consistency in reporting as per the KDHS data and evaluating trends across the years.

**Comment: Results section (2/2): We recommend that the authors present the socio-demographic characteristics of the study participants in the form of tables and/or graphs (numbers, frequencies, means, standard deviation, median, etc. depending on the characteristic and/or the outcome variable).**

**Response**: We have provided a table with the socio-demographic characteristics of the study population across the years. This is presented as Table 1 of the manuscript.

**Comment: We know that for a given variable, OR (reference modality) = 1. I suggest that the authors replace REF by 1 in each table (Tables 1, 2 and 3).**

**Response**: We are aligned with your suggestion and corrected all the REF to 1 in tables 1. 2 & 3.

**Comment: Another comment (1/1): I didn't see any appendices in the current version of the manuscript. If you have not already done so, it would be advisable to include the following: the bivariable logistic regression and the multivariable logistic regression model initial.**

**Response**: We have provided the logfiles of the STATA regression analysis models we used.

Your guidance and insights have been invaluable in enhancing the quality and rigor of our work. We appreciate your consideration of our manuscript for publication in PLOS Global Public Health and look forward to the opportunity to contribute to the dissemination of this very useful knowledge in the field. We are eagerly anticipating your endorsement of our manuscript for publication.

Best regards,

Christopher Ochieng’ Odero,

P. O. Box 51048 – 00200,

Nairobi.

E: [christodero@gmail.com](mailto:christodero@gmail.com)
